# Genomic profiling of post-transplant lymphoproliferative disorders using cell-free DNA

**DOI:** 10.1186/s13045-023-01500-x

**Published:** 2023-09-14

**Authors:** Nick Veltmaat, Yujie Zhong, Filipe Montes de Jesus, Geok Wee Tan, Johanna A. A. Bult, Martijn M. Terpstra, Pim G. N. J. Mutsaers, Wendy B. C. Stevens, Rogier Mous, Joost S. P. Vermaat, Martine E. D. Chamuleau, Walter Noordzij, Erik A. M. Verschuuren, Klaas Kok, Joost L. Kluiver, Arjan Diepstra, Wouter J. Plattel, Anke van den Berg, Marcel Nijland

**Affiliations:** 1 Department of Hematology, University Medical Centre Groningen, University of Groningen, Groningen, The Netherlands; 2 Department of Pathology and Medical Biology, University Medical Centre Groningen, University of Groningen, Groningen, The Netherlands; 3 Medical Imaging Center, Department of Nuclear Medicine and Molecular Imaging, University Medical Centre Groningen, University of Groningen, Groningen, The Netherlands; 4 Department of Genetics, University Medical Centre Groningen, University of Groningen, Groningen, The Netherlands; 5 Department of Hematology, Erasmus Medical Centre Rotterdam, Rotterdam, The Netherlands; 6 Department of Hematology, Radboud University Medical Center Nijmegen, Nijmegen, The Netherlands; 7 Department of Hematology, University Medical Center Utrecht, Utrecht, The Netherlands; 8 Department of Hematology, Leiden University Medical Center, Leiden, The Netherlands; 9 Department of Hematology, Amsterdam University Medical Center, Amsterdam, The Netherlands; 10 Department of Pulmonology, University Medical Centre Groningen, University of Groningen, Groningen, The Netherlands

**Keywords:** Post-transplant lymphoproliferative disorder, Cell-free DNA, Genomic profiling, Liquid biopsy, Epstein–Barr virus, Copy number variation, Single nucleotide variants, Low-coverage whole genome sequencing, Next generation sequencing

## Abstract

Diagnosing post-transplant lymphoproliferative disorder (PTLD) is challenging and often requires invasive procedures. Analyses of cell-free DNA (cfDNA) isolated from plasma is minimally invasive and highly effective for genomic profiling of tumors. We studied the feasibility of using cfDNA to profile PTLD and explore its potential to serve as a screening tool. We included seventeen patients with monomorphic PTLD after solid organ transplantation in this multi-center observational cohort study. We used low-coverage whole genome sequencing (lcWGS) to detect copy number variations (CNVs) and targeted next-generation sequencing (NGS) to identify Epstein-Barr virus (EBV) DNA load and somatic single nucleotide variants (SNVs) in cfDNA from plasma. Seven out of seventeen (41%) patients had EBV-positive tumors, and 13/17 (76%) had stage IV disease. Nine out of seventeen (56%) patients showed CNVs in cfDNA, with more CNVs in EBV-negative cases. Recurrent gains were detected for 3q, 11q, and 18q. Recurrent losses were observed at 6q. The fraction of EBV reads in cfDNA from EBV-positive patients was 3-log higher compared to controls and EBV-negative patients. 289 SNVs were identified, with a median of 19 per sample. SNV burden correlated significantly with lactate dehydrogenase levels. Similar SNV burdens were observed in EBV-negative and EBV-positive PTLD. The most commonly mutated genes were *TP53* and *KMT2D* (41%), followed by *SPEN*, *TET2* (35%), and *ARID1A*, *IGLL5*, and *PIM1* (29%), indicating DNA damage response, epigenetic regulation, and B-cell signaling/NFkB pathways as drivers of PTLD. Overall, CNVs were more prevalent in EBV-negative lymphoma, while no difference was observed in the number of SNVs. Our data indicated the potential of analyzing cfDNA as a tool for PTLD screening and response monitoring.

**Supplementary Information:**

The online version contains supplementary material available at 10.1186/s13045-023-01500-x.

**To the editor**

Post-transplant lymphoproliferative disorder (PTLD) is a major complication after solid organ transplantation (SOT) [1]. While immunosuppressive therapy has been associated with early Epstein-Barr virus (EBV) driven PTLD, late-onset PTLD often lack EBV and have more genomic aberrations [2–4]. Diagnosis of PTLD is challenging due to its variable presentation. Serial monitoring of plasma EBV DNA levels and [18F]FDG PET/CT have limited sensitivity and specificity [5–7]. Based on recent successes in other B-cell lymphomas, analysis of plasma derived cell-free DNA (cfDNA) offers a promising minimally invasive approach for PTLD detection and disease monitoring [8, 9].

We investigated the feasibility of genomic profiling of PTLD in 17 patients with monomorphic PTLD by cfDNA analysis. Copy number variations (CNVs) were detected using low-coverage whole-genome sequencing (lcWGS). Targeted next-generation sequencing (NGS) was used for identifying EBV DNA load and somatic single nucleotide variants (SNVs) in cfDNA using a targeted panel including the EBV BamHI-W repeat region and *LMP1*, as well as the coding regions of 72 genes commonly mutated in B-cell lymphoma. SNVs and small insertions and deletions (indels) were called using an in-house pipeline. A detailed description of materials and methods can be found in Additional file 1.

## Findings

The median age of the patients was 55 years (range 13–74). Median time between SOT and PTLD was 95 months (range 2–338). Most patients had stage IV disease (n = 13, 76%) with a median metabolic tumor volume (MTV) of 302 mL (range 5–2070 mL). Lactate dehydrogenase (LDH) levels ranged from 210 to 5068 (Additional file 1: Table S1). PTLD tissue was EBV-positive in 7 out of 17 patients (41%). EBV copies in plasma quantified by qPCR were elevated (> 5000 copies/mL) in 6 out of 15 (40%) evaluable patients.

The mean cfDNA yield of patients with PTLD (666 ng/mL, range 3–6049) was significantly higher compared to controls (21 ng/mL, range 6–54; *p* = 0.01) (Additional file 1: Figure S2A), with the observed range of cfDNA levels in PTLD patients being consistent with observations in other malignancies [10]. In PTLD patients a moderate correlation was observed between cfDNA levels and MTV (ρ = 0.53, *p* = 0.036) and LDH levels (ρ = 0.57, *p* = 0.019) (Additional file 1: Figures S2B-C).

**Fig. 1:**
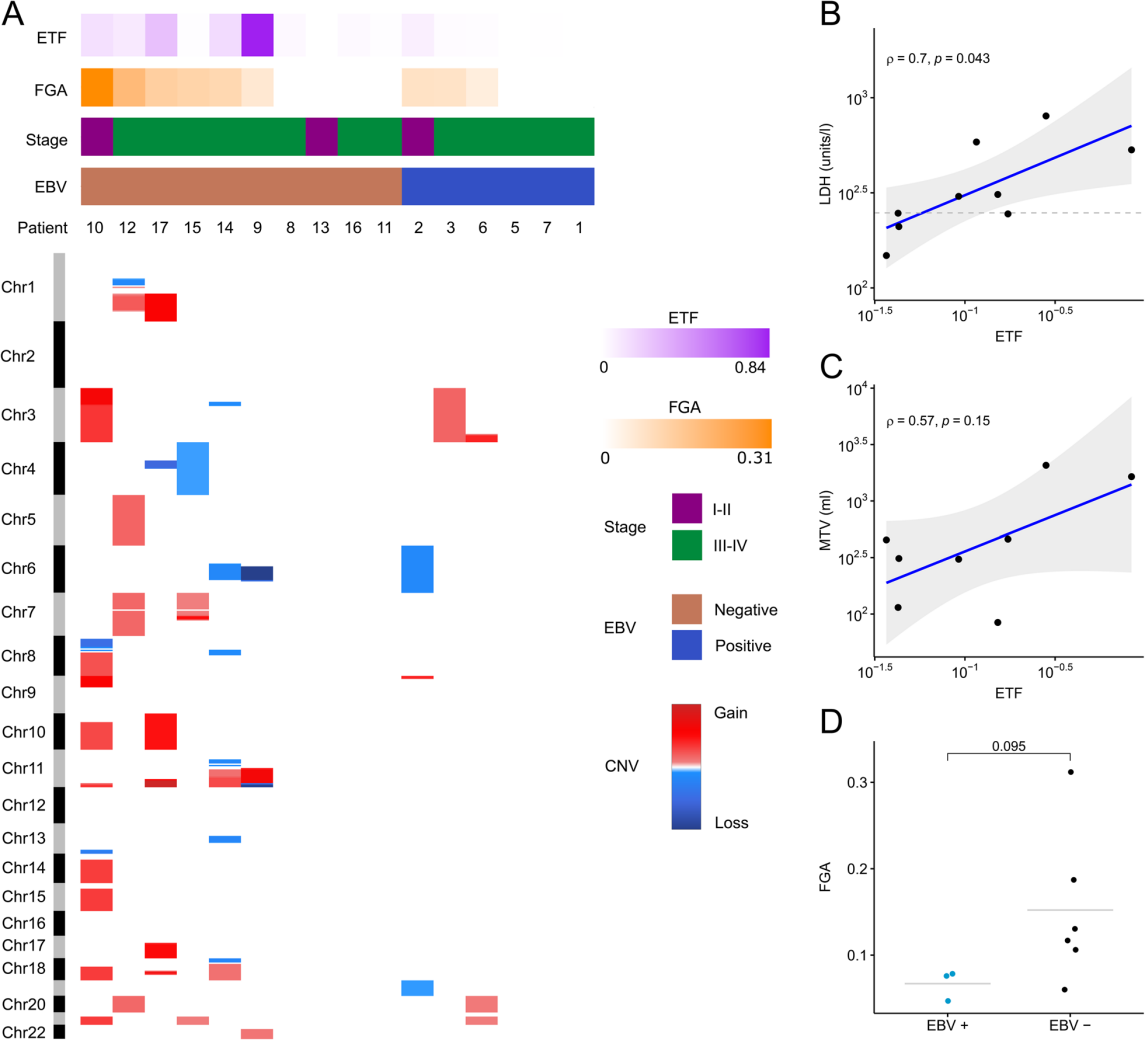
Copy number aberrations found in cell-free DNA from PTLD patients. **A** Overview of copy number variations (CNVs) for individual PTLD patients, grouped by Epstein–Barr virus (EBV) status and sorted on fraction of genome altered (FGA). Chromosomal regions with gains are indicated in red and losses in blue. **B** A significant correlation between estimated tumor fraction (ETF) and lactate dehydrogenase (LDH) was observed. Dashed line represents cut-off value at 248 U/L discriminating clinically elevated LDH from normal LDH value. **C** Correlation between ETF and metabolic tumor volume (MTV). In panels **B** and **C**, the grey areas around the regression lines represent 95% CI and the Spearman coefficient is indicated with ρ. **D** FGA in PTLD patients categorized by Epstein–Barr virus (EBV) status, as determined by EBER-ISH, shows that EBV-negative patients have a higher FGA, although insignificant according to Wilcoxon Signed Rank test. Only PTLD samples with CNVs (FGA > 0) are shown in **B–D**

CNV analysis failed in 1 patient due to insufficient sequencing reads. CNVs were detected in cfDNA in 9 out of the 16 (56%) patients. The most frequent gains involved 3q, 11q,18q and chromosome 21, while the most frequently lost region was 6q (Fig. 1A). lcWGS analysis of matched tumor samples revealed CNVs in all 5 patients. In general, more CNVs with higher amplitudes of gains/losses were observed in tissue as compared to the matched cfDNA samples, corresponding with a lower estimated tumor fraction (ETF) in cfDNA samples compared to tissue (Additional file 1: Figure S3).

The ETF was significantly correlated to LDH levels, but not to MTV (Fig. 1B, C). The number of CNVs per patient was much higher in EBV-negative patients compared to EBV-positive patients. This resulted in a higher mean fraction of genome altered (FGA) in EBV-negative tumors compared to EBV-positive tumors (0.152 vs 0.067), although not statistically significant (*p* = 0.095) (Fig. 1D).

The median percentage of EBV reads (0.53%) was significantly higher (approximately 3-logs) in EBV-positive tumors, compared to EBV-negative tumors and controls (*p* < 0.001). No significant difference was observed between EBV-negative PTLD and controls. A percentage of EBV reads above 0.0012% was indicative of an EBV-positive PTLD at the time of diagnosis (Additional file 1: Figure S4A). Six of the seven patients with EBV-positive tumors were tested positive in the diagnostic qPCR test. We observed a concordance between qPCR results and the EBV load as determined by NGS (Additional file 1: Figure S4B).

**Fig. 2:**
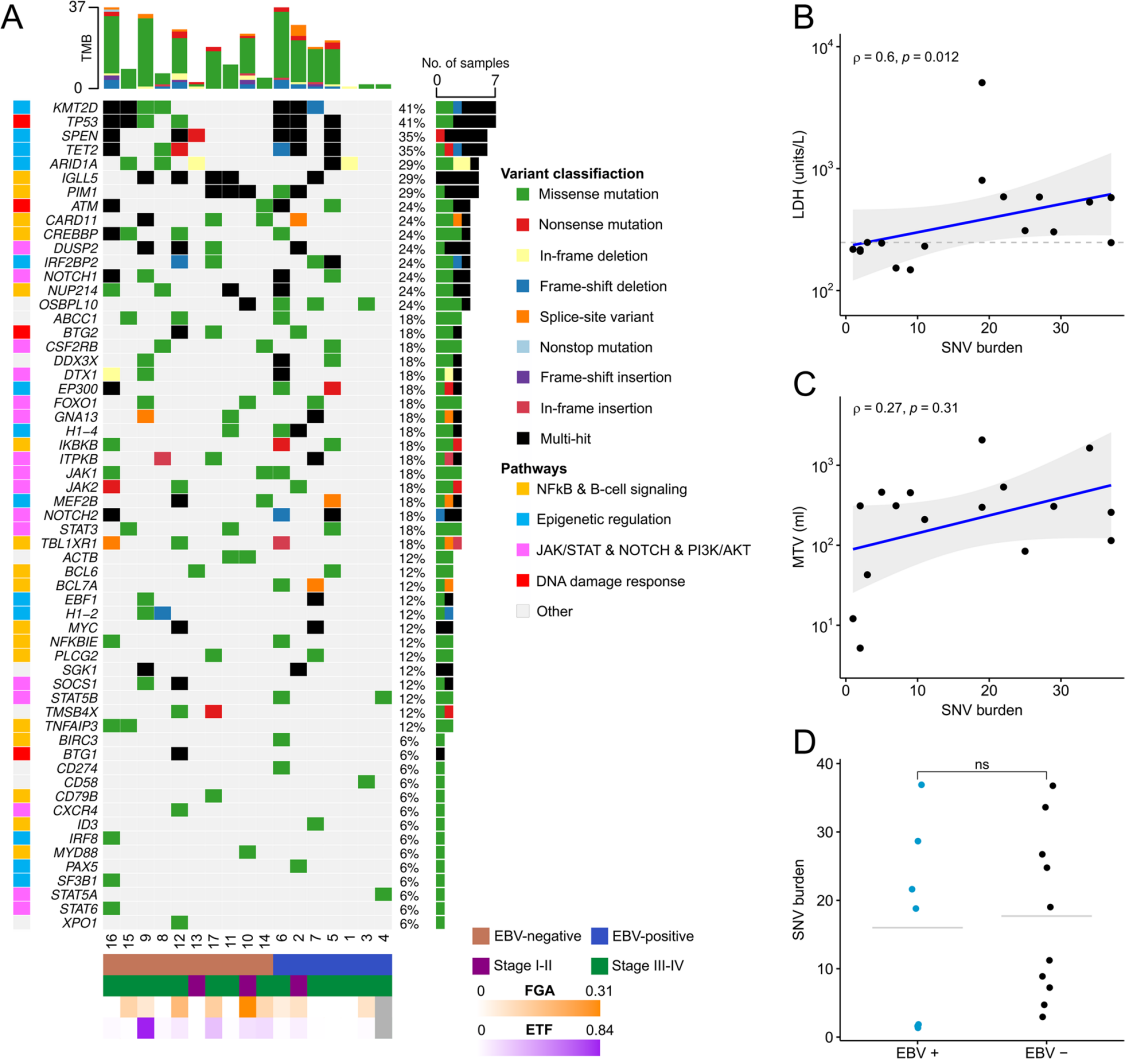
Single nucleotide variants in genes found in cfDNA of PTLD patients. **A** Waterfall plot displaying types of mutations in each plasma sample for each gene. Top- and right-side bar plots show total number of mutations found in a sample (tumor mutation burden, TMB) or a gene, respectively. Genes are sorted based on this number. Samples are grouped by Epstein Bar virus (EBV) status. EBV status, Ann-Arbor staging, Estimated tumor fraction (ETF) and fraction of genome altered (FGA) information is shown below. Pathway information per gene is indicated by color, left of the gene names. **B** A significant correlation between SNV load and lactate dehydrogenase (LDH) was observed. Dashed line represents cut-off value at 248 U/L discriminating clinically elevated LDH from normal LDH value. **C** Correlation between SNV load and metabolic tumor volume (MTV). In panels B and C, the grey areas around the regression lines represent the 95% CI and the Spearman coefficient is indicated with ρ. **D** The total number of SNVs per sample is shown, grouped by EBV status. EBV-negative samples show a slightly higher mean, though this difference is insignificant as tested by Wilcoxon Signed Rank test

A total of 289 SNVs/InDels were identified in the 17 plasma samples with a median of 19 SNVs per sample (range 1–37). The number of SNVs was significantly correlated to LDH, but not MTV (Fig. 2B, C). The most frequently mutated genes were *TP53* and *KMT2D* (7/17, 41%), *SPEN* and *TET2* (6/17 cases (35%), followed by *ARID1A*, *IGLL5* and *PIM1* (5/17, 29%) (Fig. 2A). We observed no difference in SNV burden and affected genes between EBV-negative and EBV-positive cases (Fig. 2D). Mutated genes and pathways in our study overlapped with findings of previously reported genes & pathways in PTLD [11].

In conclusion, the data of this study highlights the use of genomic profiling of plasma cfDNA analysis in patients with PTLD as a minimally invasive tool for potential screening strategies, genomic profiling and response monitoring. CNVs were successfully detected using lcWGS, while EBV status and the tumor mutational landscape could be captured using targeted NGS. EBV-negative PTLD had more CNVs compared to EBV-positive cases, suggesting a higher degree of genomic instability. Consequently, sequential EBV detection by EBV PCR and/or SNV analysis is the most suitable screening strategy for EBV-positive PTLD, while CNV and/or SNV profiling would be a good screening strategy for EBV-negative cases. The utility of SNV analyses could contribute to tumor typing at diagnosis and response assessment. This study presents the first cfDNA analysis for PTLD, with limitations of small sample size and lack of tissue biopsies in some patients. The value of ctDNA dynamics in a larger patient PTLD cohort is part of the ongoing observational NTR 7402 study [12].

### Supplementary Information

**Additional file 1**. Supplementary methods.

## Data Availability

The datasets used and/or analysed during the current study are available from the corresponding author on reasonable request.

## Abbreviations

cfDNA: Cell-free DNA
CNV: Copy number variation
EBER ISH: Epstein–Barr encoding region specific RNA in situ hybridization
EBV: Epstein–Barr virus
ETF: Estimated tumor fraction
FGA: Fraction of the genome altered
lcWGS: Low coverage whole genome sequencing
LDH: Lactate dehydrogenase
MTV: Metabolic tumor volume
NGS: Next generation sequencing
PTLD: Post-transplant lymphoproliferative disorder
qPCR: Quantitative polymerase chain reaction
R-CHOP: Rituximab, cyclophosphamide, doxorubicin, vincristine, prednisone
SNV: Single nucleotide variants
