# Supplementary material for: Genomic profiling of post-transplant lymphoproliferative disorders using cell-free DNA
Source: J Hematol Oncol. 2023 Sep 14;16:104. doi: 10.1186/s13045-023-01500-x (PMC10500745; doi:10.1186/s13045-023-01500-x)
Supplement: Supplementary file 1 — Additional file 1. Supplementary methods. [file 13045_2023_1500_MOESM1_ESM.docx]

**Contents**

[**Supplementary methods** 2](#_Toc141195912)

[Study design & patient selection 2](#_Toc141195913)

[[18F]FDG PET/CT acquisition & analysis 2](#_Toc141195914)

[Library preparation for lcWGS 3](#_Toc141195915)

[Targeted sequencing for the detection of EBV and SNVs 4](#_Toc141195916)

[Statistical analysis 5](#_Toc141195917)

[**Supplementary figures** 6](#_Toc141195918)

[Figure S1. Schematic overview of the SNV calling pipeline. 6](#_Toc141195919)

[Figure S2. Cell-free DNA yield in PTLD patients. 7](#_Toc141195920)

[Figure S3. CNVs detected by lcWGS in matched plasma and tumor tissue. 8](#_Toc141195921)

[Figure S4. EBV reads in cfDNA of PTLD patients. 9](#_Toc141195923)

[**Supplementary tables** 10](#_Toc141195924)

[Table S1: Clinical characteristics of the PTLD patients included in this study 10](#_Toc141195925)

[Table S2. Overview of the B-cell lymphoma related genes included in the targeted sequencing panel 11](#_Toc141195926)

[Table S3. Targeted sequencing quality 12](#_Toc141195927)

[**References** 13](#_Toc141195928)

# **Supplementary methods**

## **Study design & patient selection**

This observational cohort study included 17 patients with monomorphic PTLD after SOT. Patients were included based on availability of plasma samples for lcWGS and target sequencing analysis. Patients were not excluded based on demographic variables such as age and sex, transplantation type, Epstein-Barr virus (EBV) status, or the duration elapsed since the transplant procedure. Nine patients diagnosed between 2013 and 2018 at the University Medical Center Groningen (UMCG) were retrospectively included and eight patients diagnosed between 2019 and 2020 were prospectively included, of which 4 were diagnosed at UMCG, 2 at Radboud University Medical Center, 1 at Erasmus University Medical Center, and 1 at University Medical Center Utrecht. Seven plasma samples obtained from subjects without malignancies, without previous organ transplantation and not caused by an EBV infection were included as controls. These samples were derived from patients with reactive lymphadenopathy (n=4), common variable immune deficiency (n=2) and a healthy control (n=1). Diagnostic tissue biopsies were reviewed by a specialized hematopathologist (A.D.). Plasma samples were collected using EDTA tubes within 31 days of diagnosis from patients with active disease, processed within 2 hours and stored in at -80 degrees Celsius for a maximum of 84 months. Matched diagnostic formalin-fixed paraffin-embedded (FFPE) tumor tissue specimens available for this study were available for six patients, but for one patient the tissue sample was not representative. Data on tumor EBV status assessed by in situ hybridization using Epstein-Barr encoding region specific RNA (EBER) probes were retrieved from the pathology reports. Plasma EBV DNA loads determined by quantitative polymerase chain reaction (qPCR) and lactate dehydrogenase (LDH) levels were retrieved from the clinical lab reports. The study was conducted in accordance with the ethical principles of the Declaration of Helsinki and with the approval of the Medical Ethics Review Board of the University Medical Center Groningen (2018/437). Informed consent was waived for all retrospective patients and obtained for all prospectively included patients.

## **[18F]FDG PET/CT acquisition & analysis**

[^18^F]FDG PET/CT scans were performed on a Siemens Biograph mCT (Siemens Healthineers, Erlangen, Germany) according to the European Association of Nuclear Medicine (EANM) procedure guidelines for tumor imaging (1). Integrated [^18^F]FDG PET/CT images were corrected for scatter and attenuation based on CT information. Ann Arbor staging and quantification of metabolic tumor volume (MTV) were performed with [^18^F]FDG PET/CT. MTV, defined as the total metabolically active volume of the segmented tumors, was performed on a specialized software platform from Hermes Hybrid 3D (Hermes Medical Solutions AB, Stockholm, Sweden) by F.M.J.

## **Library preparation for lcWGS**

cfDNA was extracted from plasma with QIAamp Circulating Nucleic Acid kit (Qiagen, Hilden, Germany). Genomic DNA (gDNA) was extracted from FFPE tissue using the QIAamp DNA FFPE Tissue Kit (Qiagen). DNA extractions were carried out according to the manufacturer’s protocols. Library preparation was performed with Twist Library Preparation kit following the protocols by the manufacturer. Fragmentation of gDNA was done on the Covaris M220 Focused-ultrasonicator (Covaris, Massachusetts, USA). Library preparation included dA-tailing, which was followed by ligation of xGen Dual Index UMI Adapters (Integrated DNA Technologies, Leuven, Belgium). These indexed libraries were amplified using ten PCR cycles and library yields were assessed with High Sensitivity DNA Screentape (Agilent Technologies, California, USA).

lcWGS aiming at a mean coverage of 0.2X was carried out on pools consisting of 32 indexed libraries. Sequencing was performed on NovaSeq 6000 (Illumina) at GenomeScan (Leiden, Netherlands). Paired-end reads from lcWGS were mapped to human genome assembly hg19 with Burrows-Wheeler Aligner (BWA). The R package CNAclinic was used to identify genome wide CNVs and fraction of genome altered (FGA), using the circular binary segmentation algorithm. The optimal bin size was 1000kb for plasma and 500kb for tissue. IchorCNA was used to determine the fraction of ctDNA (estimated tumor fraction, ETF) in the cfDNA samples (2).

## **Targeted sequencing for the detection of EBV and SNVs**

Indexed libraries were enriched for our targeted panel encompassing 244 kb (obtained from Twist Bioscience, California, USA). The panel included the EBV BamHI-W repeat region and *LMP1*, as well as the coding regions of 72 genes commonly mutated in B-cell lymphoma (Table S2). After enrichment, libraries were PCR amplified using ten cycles. Target-enriched libraries were sequenced to a mean target coverage of 2000X for variant calling as described above. An overview of the targeted NGS sequencing quality can be found in Table S3. Paired-end reads were trimmed and subsequently mapped to the human genome assembly hg38 and to the EBV genome (NC_007605.1) using BWA. MarkDuplicates and Base Quality Score Recalibration from the GATK toolkit were applied to identify duplicate reads and to detect systematic sequencing errors, respectively. The relative percentage of EBV was calculated based on the fraction of total reads aligned to the EBV genome compared to reads aligned to hg38.

SNVs and small insertions and deletions (indels) were called using an in-house pipeline, optimized for calling somatic variants with low variant allele frequencies without the availability of matched control samples (Figure S1). In short, the pre-processed .bam files were analyzed by four different variant callers: Mutect2, LoFreq, SiNVICT and VarDict. All resulting variants were merged and filtered on read depth (RD) >100, variant allele frequency (VAF) >0.01, mutant read depth (MDP) >7 and base quality >25. Subsequently, variants were filtered using a Panel of Normals (PoN), which contained all variants called in one or more of the seven controls with any of the four callers. SNVs that overlapped with multinucleotide variants (MNVs) called by a different variant caller were removed from the final list. Finally, variants that are very likely to be sequencing artifacts (adapters, variants in repeats and/or other hard to sequence regions) were manually inspected and removed. The resulting variants were annotated using OpenCravat and filtered based on functional effect and presence in the gnomAD v3 database. Synonymous, 5’/3’ UTR-, intronic and 2kb up- and downstream variants were filtered, as well as variants with a global and european allele frequency of >0.001 in gnomAD v3. A more detailed description and scripts can be found on GitHub: <https://github.com/nickveltmaat/CircuSNV>

## **Statistical analysis**

Baseline patient characteristics were summarized graphically and through descriptive statistics. Comparison between two non-parametric variables was carried out with a Wilcoxon rank-sum test. Comparison between three or more non-parametric variables was carried out with a Kruskal-Wallis test. Correlation between variables was assessed using Spearman correlation coefficient (r). A p-value <0.05 was considered to be statistically significant. Statistical and graphical analysis were performed using open-source statistical software R with Bioconductor packages (<http://www.bio-conductor.org>) (3).

# **Supplementary figures**


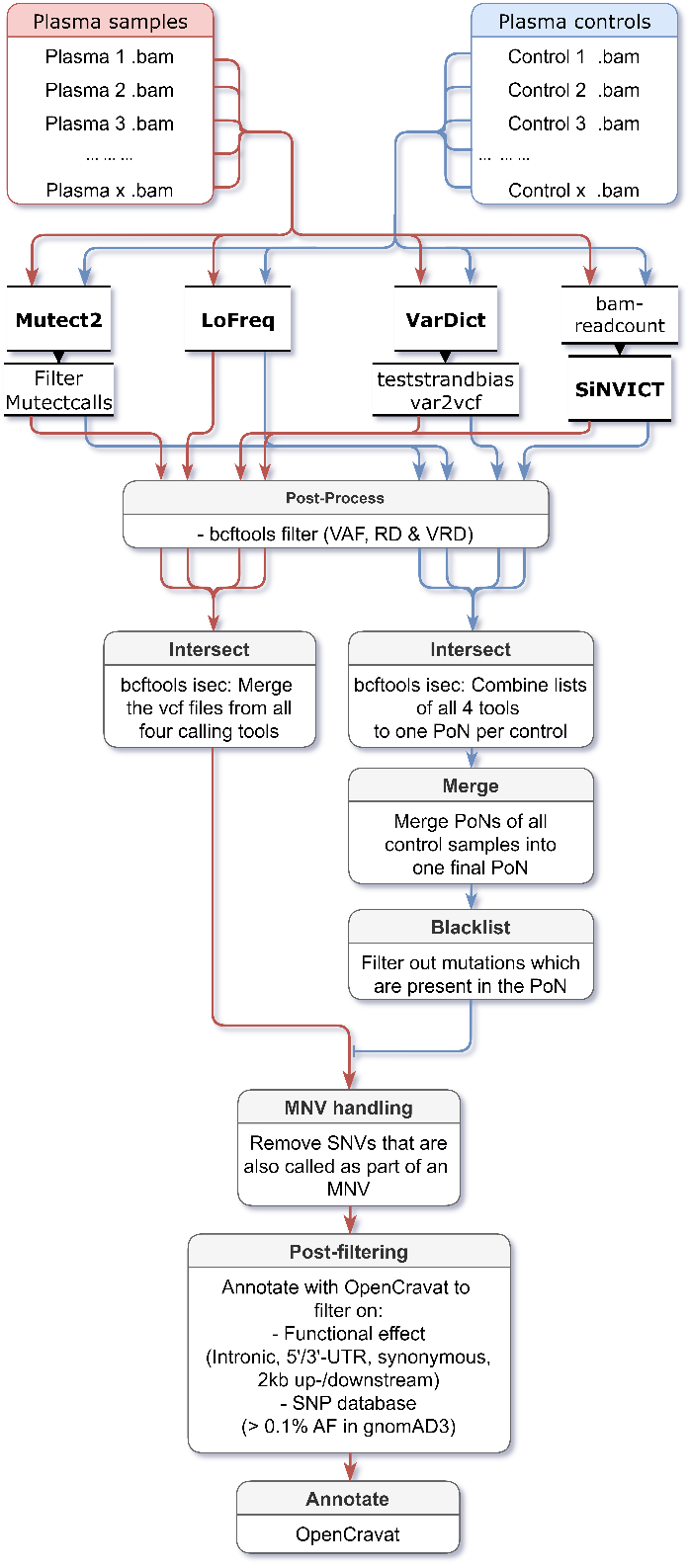


**Figure S1. Schematic overview of the SNV calling pipeline.** Each bubble represents a major step in the pipeline, indicating which tool was used for a certain process. Red arrows represent the flow of the tumor plasma samples. Blue arrows represent the flow for the control samples, for generating the panel of normals (PoN), which is eventually used to blacklist the variants found in control samples. See <https://github.com/nickveltmaat/CircuSNV> for more details.


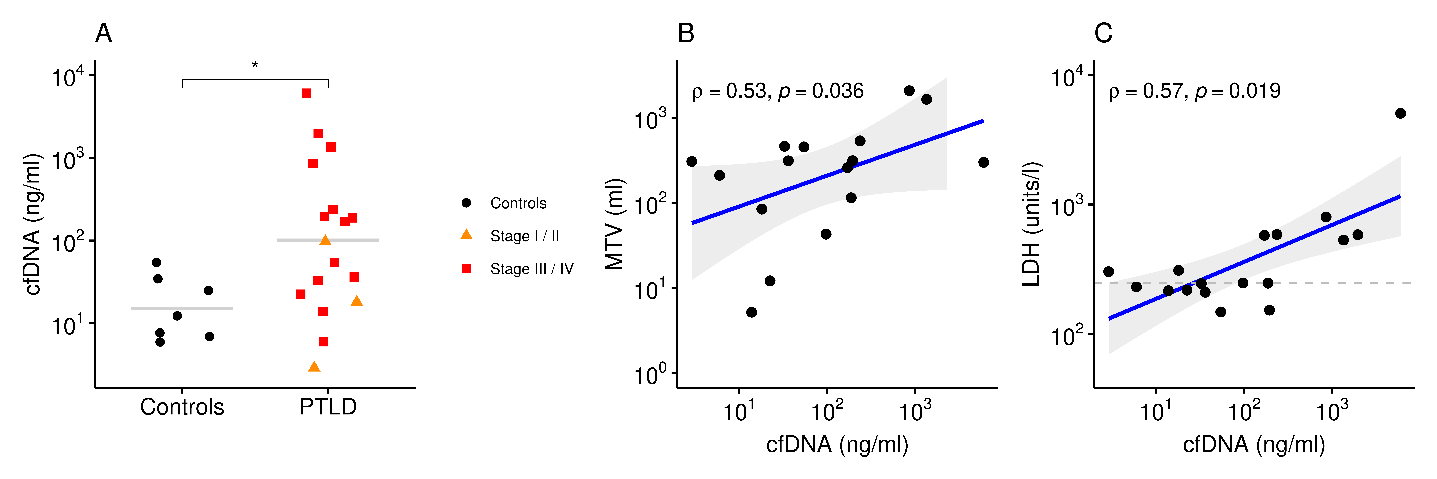


**Figure S2. Cell-free DNA yield in PTLD patients.** (A) Comparison of plasma cfDNA yield in PTLD patients shows a significantly elevated level compared to controls. Plasma levels in cfDNA from PTLD patients had a broad range (3-6049 ng/ml), which was expected. (B, C) A moderate correlation was observed between plasma cfDNA levels and metabolic tumor volume (MTV) as measured by pre-treatment [^18^F]FDG PET/CT (B), and lactate dehydrogenase (LDH) (C). In panels B and C, the grey areas around the regression lines represent 95% CI and the Spearman coefficient is indicated with ρ. The dashed line in panel C indicates cut-off (248 U/L) level used for clinically elevated LDH levels.


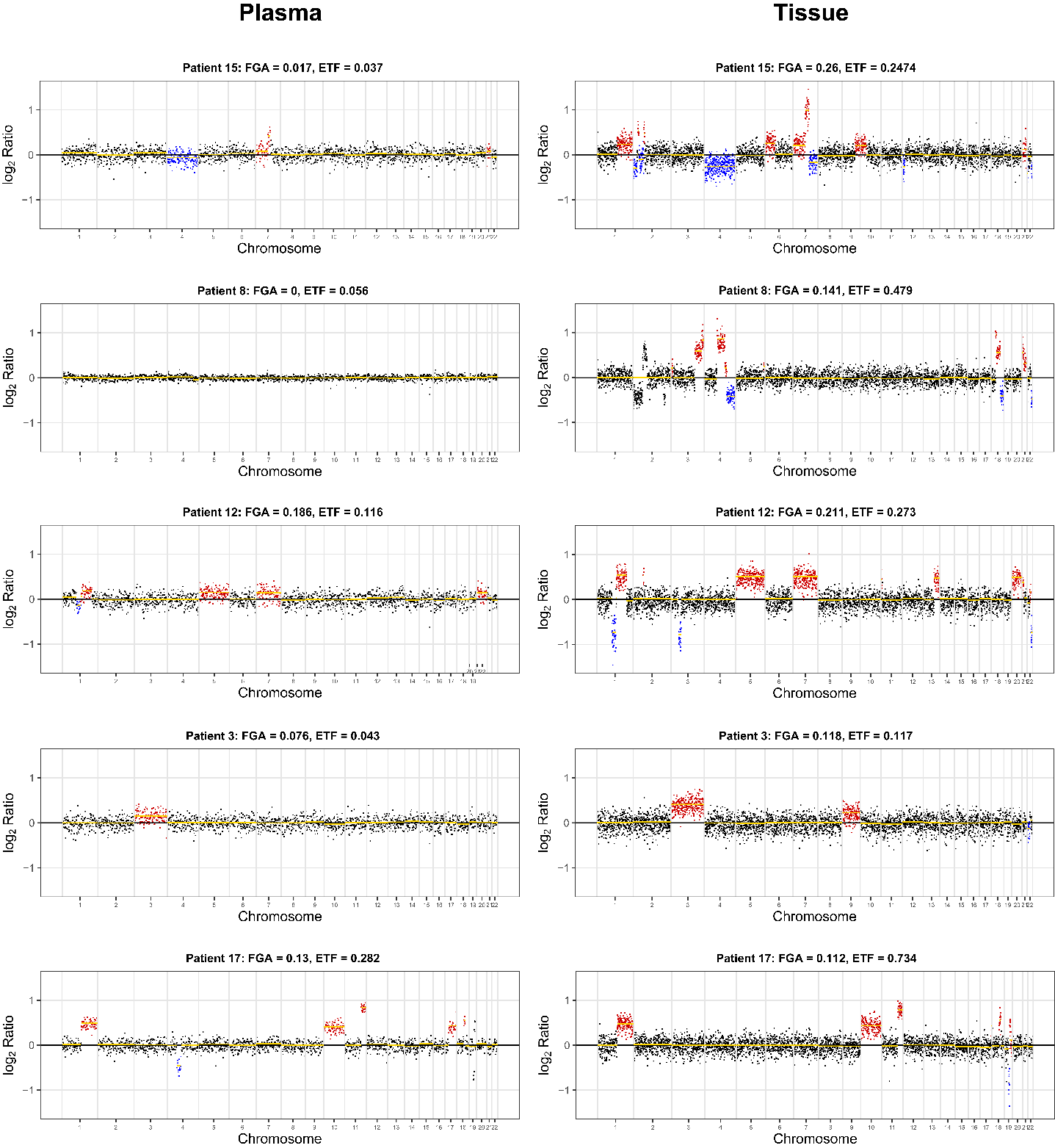


**Figure S3. CNVs detected by lcWGS in matched plasma and tumor tissue.** Copy number gains (up - red) and losses (down - blue) observed in five out of five successfully analyzed patients with matched tissue samples using lcWGS. Patient #4 had a failed lcWGS analysis for the cfDNA. Additionally, the tissue section of patient #4 was not representative, therefore this patient was left out of CNV analyses.  Patients #15, #8, #12 and #17 are EBV-negative, patient #3 is EBV-positive.

**
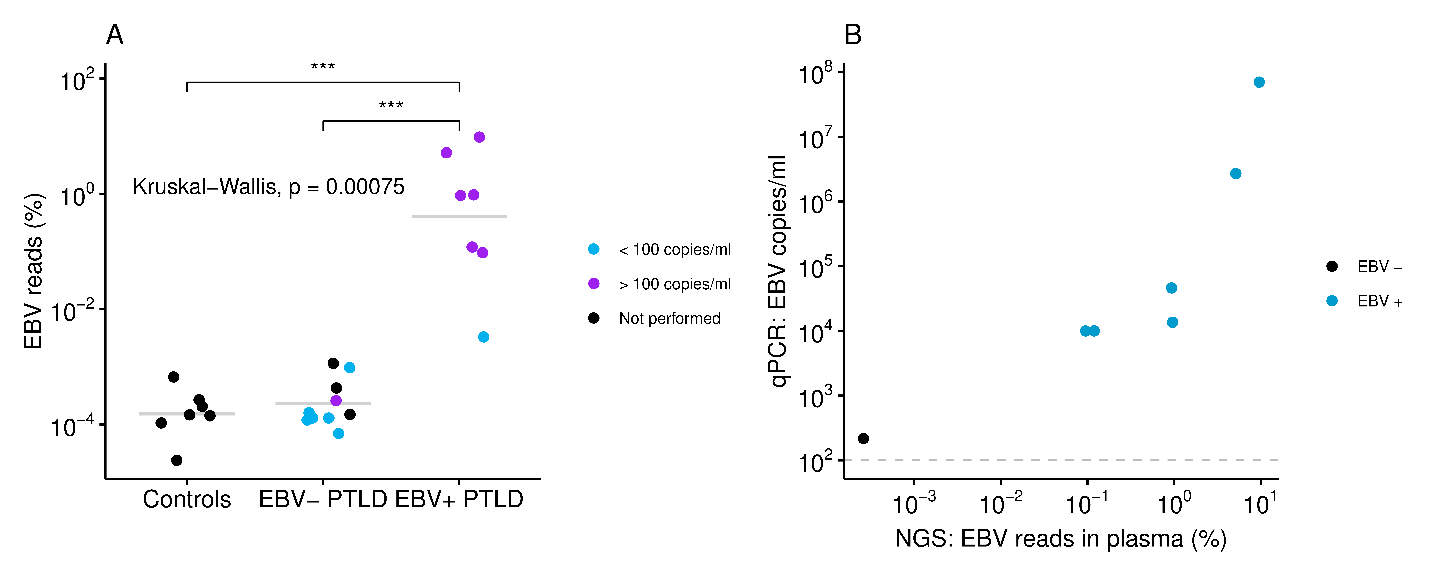
**

**Figure S4. EBV reads in cfDNA of PTLD patients.** (A) Percentage of EBV reads in cfDNA. Epstein-Barr virus (EBV) copies in plasma determined by quantitative polymerase chain reaction (qPCR) is indicated with the color of the dots. PTLD patients are grouped based on EBV classification, as determined by EBER-ISH. Statistical comparison between groups was performed using Wilcoxon Signed Rank tests where *** means p<0.001. (B) Plasma EBV detection by qPCR and targeted next generation sequencing (NGS). Percentages of EBV detected by NGS showed good concordance to EBV DNA load by qPCR in seven patients. Samples with undetected plasma EBV by qPCR (<100 copies/ml) were not shown.

# **Supplementary tables**

**Table S1:** Clinical characteristics of the PTLD patients included in this study, grouped on tissue biopsy availability and EBV status.

| **Study ID** | **EBER ISH** | **Transplanted Organ** | **Age at diagnosis** | **Gender** | **Plasma EBV qPCR (copies/ml)** | **IPI** | **Stage** | **LDH (units/L)** | **MTV (ml)** |
| --- | --- | --- | --- | --- | --- | --- | --- | --- | --- |
| **1** | + | Kidney | 73 | M | 10000 | 3 | IV | 218 | 12 |
| **2** | + | Liver | 57 | M | 10000 | 1 | II | 303 | 306 |
| **3** | + | Lung | 49 | F | 46200 | 2 | IV | 210 | 311 |
| **4** | + | Lung | 13 | F | <100 | 2 | IV | 216 | 5,2 |
| **5** | + | Lung | 55 | F | 2700000 | 3 | IV | 587 | 534 |
| **6** | + | Lung | 55 | F | 13600 | 1 | IV | 247 | 114 |
| **7** | + | Lung | 45 | M | 69700000 | 3 | IV | 5068 | 298 |
| **8** | - | Kidney | 54 | M | <100 | 1 | III | 153 | 312 |
| **9** | - | Kidney | 44 | M | <100 | 3 | IV | 532 | 1642 |
| **10** | - | Kidney | 71 | F | <100 | 2 | I | 310 | 84 |
| **11*** | - | Kidney | 44 | M | N.A. | 4 | IV | 231 | 210 |
| **12** | - | Lung | 72 | F | N.A. | N.A. | IV | 585 | N.A. |
| **13** | - | Lung | 49 | F | <100 | 1 | II | 248 | 43 |
| **14** | - | Lung | 71 | F | <100 ^(#)^ | 3 | IV | 245 | 460 |
| **15** | - | Multi-organ | 68 | F | <100 | 4 | IV | 148 | 454 |
| **16** | - | Multi-organ | 28 | F | 217 | 2 | IV | 578 | 258 |
| **17** | N.A. ^f^ | Liver | 74 | M | <100 | 4 | IV | 802 | 2070 |

^f^ EBV tumor status categorized as EBV-negative based on qPCR and targeted sequencing results.

^#^ Whole blood instead of plasma was used for qPCR.

*Received one course of Rituximab before plasma sampling.

Abbreviations: EBER ISH - Epstein-Barr encoding region specific RNA in situ hybridization; EBV - Epstein-Barr virus; IPI - International prognostic index; FFPE - formalin-fixed paraffin-embedded; LDH - lactate dehydrogenase; MTV – metabolic tumor volume; NA – not available; R-CHOP – rituximab, cyclophosphamide, doxorubicin, vincristine, prednisone

## **Table S2.** Overview of the B-cell lymphoma related genes included in the targeted sequencing panel

| ABCC1 | CIITA | IRF2BP2 | PLCG2 |
| --- | --- | --- | --- |
| ACTB | CREBBP | IRF8 | PRDM1 |
| AMELY | CSF2RB | ITPKB | REL |
| ARID1A | CXCR4 | JAK1 | SF3B1 |
| ATM | DDX3X | JAK2 | SGK1 |
| B2M | DTX1 | KMT2D | SOCS1 |
| BCL2 | DUSP2 | MAP3K14 | SPEN |
| BCL6 | EBF1 | MEF2B | STAT3 |
| BCL7A | EP300 | MYC | STAT5A |
| BIRC3 | EZH2 | MYD88 | STAT5B |
| BTG1 | FOXO1 | NFKBIE | STAT6 |
| BTG2 | GNA13 | NOTCH1 | TBL1XR1 |
| BTK | H1-2 | NOTCH2 | TET2 |
| CARD11 | H1-4 | NUP214 | TMSB4X |
| CD274 | ID3 | OSBPL10 | TNFAIP3 |
| CD58 | IGLL5 | PAX5 | TNFRSF14 |
| CD79A | IKBKB | PDCD1LG2 | TP53 |
| CD79B | IKZF1 | PIM1 | XPO1 |

## **Table S3.** Targeted sequencing quality

| **Patient ID** | **Total reads (Million)** | **Unique reads (Million)** | **Mapped reads (%)** | **Duplicate reads (%)** | **Mean Target Coverage** |
| --- | --- | --- | --- | --- | --- |
| 1 | 13.2 | 6.9 | 52,40% | 47,3% | 1078 |
| 2 | 8.9 | 3.1 | 34,20% | 65,4% | 391 |
| 3 | 11.9 | 6.8 | 57,30% | 42,4% | 1119 |
| 4 | 4.1 | 0.7 | 15,90% | 83,9% | 55 |
| 5 | 19.1 | 11.1 | 58,00% | 41,6% | 1796 |
| 6 | 8.8 | 3.4 | 38,20% | 61,4% | 463 |
| 7 | 28.8 | 16.0 | 55,30% | 44,3% | 2519 |
| 8* | 15.0 | 5.9 | NA* | 60,9% | 768 |
| 9 | 10.1 | 6.6 | 64,80% | 34,9% | 1149 |
| 10 | 12.6 | 7.5 | 58,80% | 40,9% | 1166 |
| 11 | 10.8 | 4.9 | 44,90% | 54,8% | 700 |
| 12 | 9.6 | 5.8 | 59,90% | 39,8% | 909 |
| 13 | 10.6 | 6.4 | 59,60% | 40,1% | 1037 |
| 14 | 8.3 | 4.7 | 45,80% | 43,9% | 764 |
| 15 | 13.6 | 7.8 | 56,80% | 42,8% | 1258 |
| 16 | 14.5 | 8.0 | 54,60% | 45,1% | 1272 |
| 17 | 14.3 | 8.6 | 59,50% | 40,1% | 1304 |

*Sample was downsampled to 15M reads

# **References**

1. Boellaard R, Delgado-Bolton R, Oyen WJG, Giammarile F, Tatsch K, Eschner W, et al. FDG PET/CT: EANM procedure guidelines for tumour imaging: version 2.0. Eur J Nucl Med Mol Imaging. 2015 -02;42(2):328-54.

2. Adalsteinsson VA, Ha G, Freeman SS, Choudhury AD, Stover DG, Parsons HA, et al. Scalable whole-exome sequencing of cell-free DNA reveals high concordance with metastatic tumors. Nat Commun. 2017 -11-06;8(1):1324.

3. Gentleman RC, Carey VJ, Bates DM, Bolstad B, Dettling M, Dudoit S, et al. Bioconductor: open software development for computational biology and bioinformatics. Genome Biol. 2004;5(10):R80.

stylefix
